# The Bright and the Dark Side of TGF-β Signaling in Hepatocellular Carcinoma: Mechanisms, Dysregulation, and Therapeutic Implications

**DOI:** 10.3390/cancers14040940

**Published:** 2022-02-14

**Authors:** Medine Zeynep Gungor, Merve Uysal, Serif Senturk

**Affiliations:** 1 Izmir Biomedicine and Genome Center, Izmir 35340, Turkey; zeynep.gungor@msfr.ibg.edu.tr (M.Z.G.); merve.uysal@msfr.ibg.edu.tr (M.U.); 2 Department of Genome Sciences and Molecular Biotechnology, Izmir International Biomedicine and Genome Institute, Dokuz Eylul University, Izmir 35340, Turkey

**Keywords:** TGF-β signaling, hepatocellular carcinoma, tumor suppressor, pro-tumorigenic, therapy

## Abstract

**Simple Summary:**

Transforming growth factor β (TGF-β) signaling is a preeminent regulator of diverse cellular and physiological processes. Frequent dysregulation of TGF-β signaling has been implicated in cancer. In hepatocellular carcinoma (HCC), the most prevalent form of primary liver cancer, the autocrine and paracrine effects of TGF-β have paradoxical implications. While acting as a potent tumor suppressor pathway in the early stages of malignancy, TGF-β diverts to a promoter of tumor progression in the late stages, reflecting its bright and dark natures, respectively. Within this context, targeting TGF-β represents a promising therapeutic option for HCC treatment. We discuss here the molecular properties of TGF-β signaling in HCC, attempting to provide an overview of its effects on tumor cells and the stroma. We also seek to evaluate the dysregulation mechanisms that mediate the functional switch of TGF-β from a tumor suppressor to a pro-tumorigenic signal. Finally, we reconcile its biphasic nature with the therapeutic implications.

**Abstract:**

Hepatocellular carcinoma (HCC) is associated with genetic and nongenetic aberrations that impact multiple genes and pathways, including the frequently dysregulated transforming growth factor β (TGF-β) signaling pathway. The regulatory cytokine TGF-β and its signaling effectors govern a broad spectrum of spatiotemporally regulated molecular and cellular responses, yet paradoxically have dual and opposing roles in HCC progression. In the early stages of tumorigenesis, TGF-β signaling enforces profound tumor-suppressive effects, primarily by inducing cell cycle arrest, cellular senescence, autophagy, and apoptosis. However, as the tumor advances in malignant progression, TGF-β functionally switches to a pro-tumorigenic signal, eliciting aggressive tumor traits, such as epithelial–mesenchymal transition, tumor microenvironment remodeling, and immune evasion of cancer cells. On this account, the inhibition of TGF-β signaling is recognized as a promising therapeutic strategy for advanced HCC. In this review, we evaluate the functions and mechanisms of TGF-β signaling and relate its complex and pleiotropic biology to HCC pathophysiology, attempting to provide a detailed perspective on the molecular determinants underlying its functional diversion. We also address the therapeutic implications of the dichotomous nature of TGF-β signaling and highlight the rationale for targeting this pathway for HCC treatment, alone or in combination with other agents.

## 1. Introduction

According to Global Cancer Observatory estimates, liver cancer is the sixth most common type of cancer worldwide, with 905.677 new cases in 2020 [1]. As the most prevalent form, HCC accounts for nearly 90% of all primary liver malignancies [2,3]. Currently, there is a significant dearth of disease-specific early diagnostic markers and effective therapeutic modalities for advanced HCC, which poses numerous clinical problems [4]. As the incidence and mortality rate continue to grow, the annual burden of liver-cancer-related deaths is predicted to reach 1 million by 2030 [2]. The molecular pathophysiology of HCC constitutes a complex multistep process driven by the interplay of various contextual cues involving cell extrinsic factors and cell intrinsic mechanisms, such as dysregulated intracellular signaling cascades, including the transforming growth factor β (TGF-β) signaling pathway [5,6].

The pleiotropic cytokine TGF-β and its signaling effectors play essential roles in a diverse set of spatially and temporally controlled physiological processes, ranging from development and differentiation to cell proliferation, apoptotic cell death, migration, and inflammation [7]. The molecular functions of TGF-β signaling are operated by canonical and non-canonical cascades, also known as Smad-dependent and Smad-independent, respectively [8]. Consistent with its diverse attributes in almost all cell types, dysregulation of TGF-β signaling has pathophysiological consequences, including cancer [9]. In the context of liver physiology, TGF-β exhibits potent cytostatic and proapoptotic effects in normal and premalignant hepatocytes, as well as early stage liver cancer cells [10,11]. However, during the course of HCC progression, TGF-β switches to a pro-tumorigenic signal within the tumor itself and in the tumor stroma, inducing aggressive phenotypes, such as cancer cell proliferation, epithelial–mesenchymal transition (EMT), as well as tumor microenvironment (TME) remodeling and immune evasion of cancer cells [12]. In this article, we thoroughly review the dichotomous functions of TGF-β signaling in HCC and outline the implications of its biphasic nature for therapeutic interventions. To that purpose, we first present a summary of HCC pathogenesis before elaborating in detail the molecular properties of the TGF-β pathway, with an emphasis on the canonical cascade with occasional references to the non-canonical signaling circuits. Thereafter, we focus on the mechanisms that potentially underlie the functional dysregulation of TGF-β in HCC, aiming to offer insights into how the bright side of this pathway turns dark. Finally, we explore the existing and emerging treatment modalities targeting TGF-β signaling in this lethal malignancy.

## 2. Molecular Characteristics of Hepatocellular Carcinoma

HCC is an inflammation-associated cancer that frequently arises in the context of chronic liver dysfunction. The molecular etiology and pathophysiology of HCC is complex and multifactorial [13]. The major risk factors are viral infections (e.g., hepatitis B and hepatitis C), excessive alcohol consumption, aflatoxin B1 exposure, and metabolic syndromes [14]. The long-term manifestations of these etiological factors involve increased genome-wide copy number aberrations, gene mutations, epigenetic and transcriptomic alterations, and dysregulations in multiple signaling pathways [15,16]. Inactivation of the bona fide p53/pRb tumor suppressor pathway through somatic mutations has been documented as an early molecular event in more than 30% of tumors [17]. Studies have also reported oncogenic activation of WNT/β-catenin signaling pathway as a core driver event, caused by gain-of-function mutations in β-catenin or inactivating mutations of the WNT regulators Axin1 and APC [2]. Aberrant reactivation of telomerase activity, primarily caused by recurrent somatic mutations within the TERT gene promoter, is also recognized as a frequent driver event in hepatocarcinogenesis [18]. Such mutations create de novo consensus binding motifs for transcription factors, which enhance the expression of telomerase [19,20]. While these mutations are not found in cirrhotic livers, they are common in HCC tumors, suggesting that the telomerase may act as a gatekeeper in HCC evolution [21]. Homozygous deletions are less frequent in HCC. Recurrent deletions usually affect tumor suppressor genes, including the pRb regulators CDKN2A and CDKN2B (encode p16^INK4a^ and p15^INK4b^, respectively) and PTEN, which counteracts PI3K/Akt signaling by degrading its product, phosphatidylinositol (3,4,5) trisphosphate (PIP3) [22,23]. Recent studies have identified inactivating mutations in chromatin-remodeling complexes (e.g., ARID1A, ARID2, SETD2, and BRD7), highlighting the presence of an altered epigenetic landscape in HCC [24]. Focal amplifications in the FGF19-CCND1 locus, MET, VEGFA, and MYC may also contribute to hepatocarcinogenesis by oncogenic activation of crucial survival pathways [2]. Much like these cellular pathways, TGF-β signaling is strongly implicated in HCC. However, recurrent mutations in core signaling components are rarely found in tumors. Yet, studies have demonstrated higher rates of damaging mutations for the ACVR2A gene, which encodes an activin type 2 receptor that belongs to the TGF-β receptor superfamily [25].

## 3. Overview of the TGF-β Superfamily

The TGF-β superfamily, with 32 members identified in humans, is the largest family of secreted cytokines in vertebrates and invertebrates. The family members comprise highly conserved signaling ligands. Sequence and structure similarities define two ligand subfamilies. TGF-β-like proteins are the first subfamily, which includes TGF-βs, activins and inhibins, whereas the bone morphogenetic proteins (BMPs) and growth and differentiation factors (GDFs) constitute the second subfamily [26]. The cytokines of this superfamily are secreted as precursor proteins and undergo proteolytic cleavage by endoproteases to liberate and activate mature disulfide-linked dimers. The active ligands from both subfamilies classically relay signals from the cell membrane to the nucleus through type I and type II transmembrane serine/threonine kinase receptors and receptor-activated intracellular mediators. Five type II receptors and seven type I receptors, also referred to as activin receptor-like kinases (ALK1 through 7), exist in humans. The type II receptor family comprises the TGF-β type II receptor (TGFβRII), activin type II receptors (ActRIIA and ActRIIB), BMP type II receptor (BMPRII), and anti-Müllerian hormone type II receptor (AMHRII) [27,28]. Despite some promiscuous cross-reactivity, each member of this cytokine superfamily has a discrete combination of receptors for signal initiation. Besides, they use distinct downstream components for signal propagation and biological responses. TGF-β superfamily members regulate gene expression via receptor-regulated Smad transcription factors (R-Smads), and the common mediator Smad (co-Smad) serves as a shared partner of the R-Smads. In essence, TGF-β family members radiate signals via the R-Smads, Smad2, and Smad3, whereas BMP signaling is transmitted by Smad1, Smad5, and Smad9 (historically known as Smad8), all of which form a heterocomplex with the co-Smad, Smad4. The Smad complexes are subsequently translocated into the nucleus to activate or repress the transcription of a large set of target genes in cooperation with other transcriptional comodulators [29]. TGF-β signaling is counteracted by the activities of Smad6 and Smad7, members of a third Smad subfamily, known as the inhibitory Smads (I-Smads) [30]. As this review focuses on the TGF-β pathway, the founding member of the large superfamily, we primarily highlight the general knowledge on the molecular functions and mechanisms of this signaling cascade with occasional mentions to other family members.

## 4. The Molecular Mechanisms of TGF-β Signaling

The TGF-β branch of the large superfamily can be activated by three ligands: TGF-β1, TGF-β2, and TGF-β3 (collectively referred to as TGF-β), which are encoded by distinct genes in the mammalian genome. TGF-β1 is the most abundant and prototypical isoform and can be secreted by virtually all types of cells. Importantly, the TGF-β ligand is secreted in the form of a trimolecular latent complex composed of three proteins: TGF-β, the latency-associated peptide (LAP), and the latent TGF-β binding protein (LTBP) [31]. Following furin-mediated cleavage, the disulfide-linked dimers of TGF-β and LAP are linked by noncovalent bonds to form the small latent complex. In many cell types, this complex is covalently crosslinked to microfibril-associated LTBP, forming the large latent complex. Upon secretion, LTBP locally tethers inactive TGF-β to fibronectin and fibrillin in the extracellular matrix (ECM). The efficient activation of the latent TGF-β complex, a critical event in modulating TGF-β functions, can be orchestrated by extracellular serine proteases (e.g., plasmin and cathepsin D), thrombospondins (e.g., TSP-1), matrix metalloproteinases (e.g., MMP9 and MMP14), and integrins [32]. After being released from LAP or the latent complex, the active TGF-β molecules are now exposed to their cell surface receptors [33]. Ligand isoforms have overlapping receptor usage and they transduce signaling through a single type II receptor (TGFβRII). The binding of active TGF-β dimers to homodimeric TGFβRII induces the assembly of a heterotetrameric TGF-β receptor complex, upon which the constitutively active TGFβRII recruits and transphosphorylates the type I receptor (TGFβRI, also known as ALK5) to initiate intracellular signaling [34]. There is also the type III receptor (TGFβRIII), a membrane-anchored proteoglycan coreceptor that exists in many cell types. Despite not being directly engaged in signal transduction, TGFβRIII facilitates ligand presentation to TGFβRII [35]. Once activated, TGFβRI transmits regulatory instructions to the cytosol that can operate the canonical and non-canonical signaling branches, also known as Smad-dependent and Smad-independent, respectively [36].

**Figure 1:**
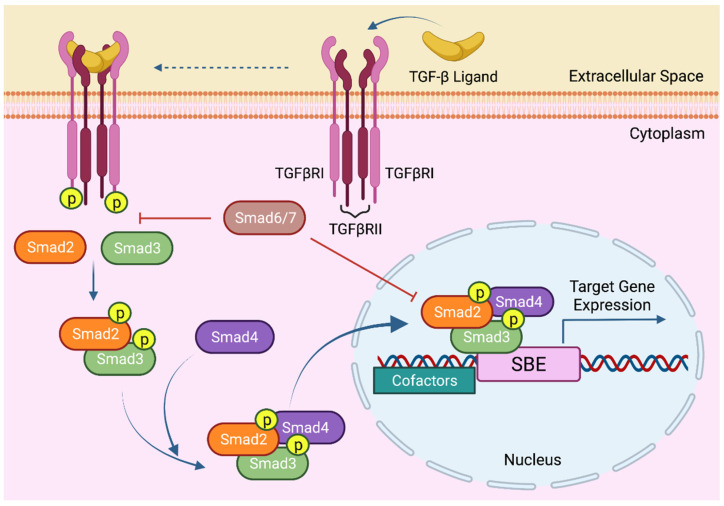
Canonical TGF-β Signaling. The TGF-β signaling pathway initiates with the binding of the TGF-β ligand to TGFβRII. Activated TGFβRII forms a complex with and phosphorylates TGFβRI. Upon phosphorylation by TGFβRI, R-Smads, Smad2, and Smad3, form a transcriptional complex with the co-Smad, Smad4. This complex then translocates into the nucleus, associates with DNA by cooperating with various cofactors, and regulates target gene expression. Smad6 and Smad7, the I-Smads, function as negative regulators of TGF-β/Smad signaling.

In the canonical TGF-β pathway, the signal is transferred into the nucleus via intracellular Smad molecules. Structurally, both R-Smads, Smad2 and Smad3, and the co-Smad, Smad4, contain two conserved mad homology (MH) domains and a proline rich linker region that separates these globular domains. The N-terminal MH1 domain is mainly responsible for DNA binding, whereas the C-terminal MH2 domain modulates the TGFβRI and R-Smad interaction, oligomeric Smad complex formation, and Smad interactions with other proteins [37]. The linker region, on the other hand, is frequently implicated in crosstalk with other signaling pathways [38]. When activated, TGF-β receptors experience conformational changes that permit R-Smads to be recruited and phosphorylated at their C-terminal SSXS motif by TGFβRI. Once phosphorylated, R-Smads dissociate from the receptor, form a heterotrimeric complex with Smad4, and translocate into the nucleus to elicit a sequence-specific transcriptional regulation of more than 500 genes [39]. Importantly, the sequence recognized by Smad3 and Smad4 is an 8 bp palindromic (GTCTAGAC) Smad-binding element (SBE). Of particular relevance, the most common alternatively spliced form of Smad2 does not bind DNA due to 30 additional amino acids positioned within the MH1 domain that interfere with DNA interaction. Canonical Smad signaling is spatiotemporally fine-tuned by the inhibitory Smads (I-Smads), Smad6 and Smad7 [40,41]. Much like the R-Smads and Smad4, the I-Smads have a conserved MH2 domain that facilitates the interaction with R-Smads and TGFβRI, thus preventing R-Smad recruitment and phosphorylation [42,43] (Figure 1).

The cellular and molecular effects of TGF-β signaling are multifaceted and context-dependent. Many lines of evidence have proven that the diversity of TGF-β-mediated transcriptional regulation is dictated by contextual availability and the direct cooperation of Smad-interacting DNA binding transcription factors. Studies have identified several partner transcriptional comodulators that function in conjunction with the Smad proteins, including intracellular receptors c-Myc, Ski/SnoN, AP-1, p300/CBP, and multiple zinc finger and the basic helix-loop-helix transcription factors [44].

**Figure 2:**
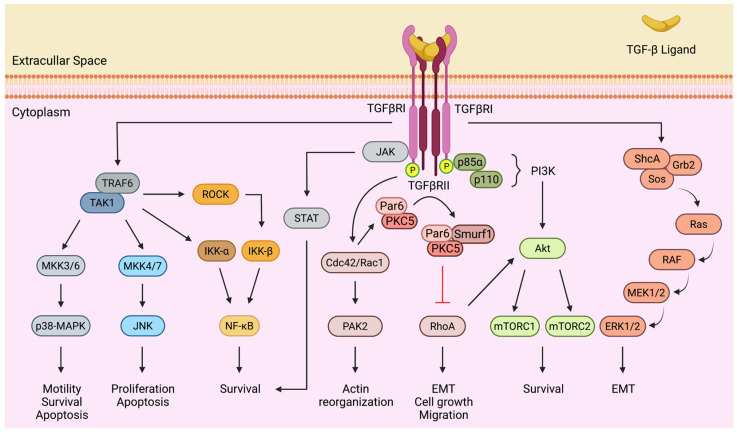
Non-canonical TGF-β Signaling. TGF-β can activate various Smad-independent pathways, including Jthe AK/STAT, PI3K/Akt, MAPK pathways (ERK, JNK, and p38-MAPK), NF-κB, or small GTPases, such as RhoA, Rac1, and Cdc42. The crosstalk between TGF-β and other signaling circuits regulates multiple biological processes, including transcription, motility, survival, and apoptosis.

Adding to the spatiotemporal dynamics of canonical Smad signaling, the complexity of TGF-β actions is further enriched by non-canonical crosstalk with other signal transduction networks. Research in the last two decades has shown that activated TGF-β receptors can transduce signals to various members of mitogen-activated protein kinases, MAPKs (i.e., extracellular signal-regulated kinases, ERKs; c-Jun N-terminal kinases, JNKs; and p38-MAPKs), nuclear factor kappa B (NF-κB), phosphatidylinositol-3-kinase (PI3K)/Akt, Janus kinases-signal transducer and activator of transcription proteins (JAK-STATs), and Rho family GTPases, such as RhoA, Rac1, and Cdc42 [45]. The activation of these pathways appears to be Smad-independent and mediated by the phosphorylation or direct interaction of pathway components with TGF-β receptors. Beyond this, the intrinsic tyrosine phosphorylation activity of the TGF-β receptor kinase domains is considered to be a critical determinant of the complex interplay between TGF-β and the non-canonical signaling cascades [46] (Figure 2).

TGF-β is a robust activator of the ERK pathway. Upon activation by ligands, TGFβRI recruits and directly phosphorylates ShcA on both tyrosine and serine residues, leading to a complex formation with Grb2 and Sos, which results in a Ras-mediated sequential activation of RAF, MEK1/2, and ERK1/2 [47]. TGFβRI is also known to induce the Smad-independent constitutive activation of the JNK and p38-MAPK cascades by the MAP kinase kinases (MKKs), particularly MKK4/7 and MKK3/6, respectively. Further upstream, TGF-β-activated kinase 1 (TAK1), a member of the MAP3K serine/threonine kinase family, phosphorylates and activates the MKKs [46]. TAK1 activation by TGF-β centers on the Lys63-linked polyubiquitylation of TAK1 by the TGFβRI-interacting E3 ubiquitin ligase TRAF6 [48]. The TRAF6-TAK1 axis similarly mediates the phosphorylation and activation of IκB-kinase (IKK) α and partakes in the crosstalk of TGF-β with NF-kB signaling [34]. Alternatively, TAK1 may flux signals to NF-kB by activating Rho-associated kinase (ROCK), a downstream effector of RhoA that phosphorylates and activates IKK-β [49]. Similar outcomes may derive from direct and indirect crosstalk between the PI3K/Akt pathway and Smad-independent TGF-β signaling. In response to TGF-β stimulation, TGF-β receptors form a complex with p85α, the regulatory subunit of PI3K, leading to the activation of Akt and downstream effectors, such as mTORC1 and mTORC2 [50,51]. Interestingly, TGF-β-mediated PI3K/Akt activation requires, at least in some settings, the TRAF6-dependent Lys63-linked polyubiquitylation of p85α [51]. The ubiquitination of Akt by TRAF6 is also required for its membrane localization and phosphorylation by PI3K [52].

Notable research has focused on the Rho GTPase-selective execution of TGF-β–mediated responses, including cytoskeletal remodeling, EMT, and cell motility, in normal and neoplastic cells [53]. In addition to RhoA, Cdc42 and Rac1 can also be activated by TGF-β receptors. In fibroblasts and epithelial cells, TGF-β-activated Cdc42 promotes p21 (Rac1) activated kinase 2 (PAK2) activation and orchestrates actin cytoskeleton meshwork organization [54]. Despite being repeatedly shown to increase the activity of Rho GTPases, TGF-β has also been reported to negatively regulate RhoA signaling in the context of EMT. Extensive data reveals that this is mediated by the TGFβRII-dependent phosphorylation of Par6, a cell polarity protein localized to tight junctions, which recruits the E3 ubiquitin ligase Smurf1. Upon assembly, the Par6-Smurf1 complex targets RhoA for localized ubiquitination and degradation, which promotes the dissolution of tight junctions, an early event in TGF-β-induced EMT [55].

Mounting evidence suggests that TGF-β may activate JAK-STAT signaling. Studies have shown that TGF-β stimulation can trigger IL-6 expression and the ensuing activation of Stat3 in several cell types, including hepatocytes and hepatic stellate cells [56]. In some cases, this crosstalk is explained by a secondary and delayed effect of the cytokine release induced by Smad-dependent signaling [57]. Intriguingly, similar events were documented in the development of targeted therapy resistance in lung cancer [58]. Finally, few studies have shown the rapid activation of Stat3 in a Smad-independent manner, through a direct and constitutive interaction between JAK1 and TGFβRI [56].

The R-Smads can be reciprocally modulated by intracellular kinases, including PI3K/Akt, p38-MAPK, JNK, ERK, glycogen synthase kinase-3β (GSK-3β), and the cyclin-dependent kinases (CDKs) [54]. While the details remain to be ascertained, there is already a great deal of knowledge regarding the molecular mechanisms by which Smad signaling crosstalks with these pathways [59,60]. As will be discussed further on, this mode of regulation generally involves the phosphorylation of R-Smads at multiple serine/threonine residues located in their linker regions. By that means, these kinases impact the stability, subcellular localization, and transcriptional activity of R-Smads and, hence, modulate TGF-β responses [38].

## 5. The Role of TGF-β Signaling in HCC

The functions of TGF-β signaling in HCC are somewhat paradoxical. While acting as a tumor suppressor by stimulating cell cycle arrest and cellular senescence, as well as evoking apoptosis and autophagy in the early stages of tumor progression, TGF-β is redirected away from thwarting cell survival and is found instead to switch to a promoter of tumor progression in the late stages, especially when cancer cells acquire resistance to its cytostatic and apoptotic effects. TGF-β signaling at this stage confers aggressive traits associated with cell survival, EMT, invasiveness and metastasis [59,61]. Furthermore, TGF-β also promotes tissue microenvironment remodeling and causes phenotypic and functional immune alterations, reducing the permissiveness of tumor cells to immune suppression and further contributing to its pro-tumorigenic effects [62]. Given the breadth of its molecular attributes in the regulation of multiple cell-biological processes associated with the maintenance of tissue homeostasis, it is not surprising that perturbation of TGF-β signaling, in both the canonical and non-canonical branches, constitutes a causative factor for neoplastic progression. From here on, we will reference the pertinent research on the biphasic and opposing properties of TGF-β signaling in HCC, with the motivation of providing insights towards a better understanding of the molecular mechanisms that turn a once bright side dark (Table 1).

### 5.1. Tumor Suppressor Role

**Figure 3:**
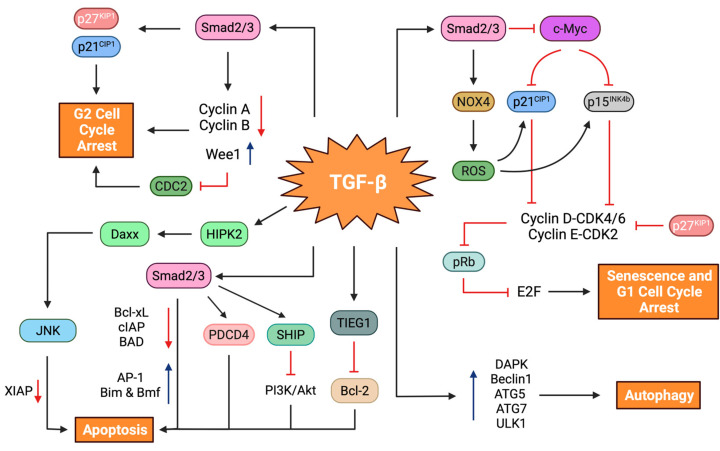
Tumor suppressor functions of TGF-β signaling. TGF-β inhibits cell proliferation and induces cellular senescence by increasing the expression of the CDK inhibitors p21^CIP1^ and p15^INK4b^, with a reciprocal decrease in hypophosphorylated pRb and c-Myc levels. This process generally requires NOX4-mediated ROS production. TGF-β can also induce G2 cell cycle arrest by upregulating p21^CIP1^ and p27^KIP1^, accompanied by a decrease in cyclin A and cyclin B levels and an increased activity of Wee1 kinase. In addition, TGF-β stimulates apoptosis by modulating several effector molecules and signaling circuits, including Bcl-xL, Bim, Bmf, cIAP, XIAP, AP-1, and PDCD4, and the PI3K/Akt cascade. TGF-β-induced autophagy involves the activation of several autophagy-related molecules, such as DAPK, Beclin1, ATG5, ATG7, and ULK1.

Arguably the most fundamental trait of TGF-β signaling is its cytostatic and proapoptotic functions in normal cells, including non-neoplastic hepatocytes. Furthermore, these responses are retained, to some extent, as tumor-suppressive actions in premalignant and malignant hepatocytes. In subsequent sections, we discuss the molecular mechanisms through which TGF-β governs decisions on suppressing cell proliferation and survival. To that end, we place a special emphasis on cell cycle arrest, cellular senescence, autophagy, and apoptosis (Figure 3).

#### 5.1.1. Cell Cycle Arrest

The orderly progression of the cell cycle is governed by a family of CDKs, whose catalytic activities are tightly regulated by their biomolecular interactions with cyclin partners and CDK inhibitors [63]. As revealed in previous studies by us and others, TGF-β elicits cytostatic G1 arrest in HCC cells, mainly through two synergistic mechanisms, (1) the increased expression of specific CDK inhibitors, and (2) the repression of growth promoting factors [64,65]. At the core of CDK inhibition is the augmented expression of tumor suppressor CDK inhibitors, such as p15^INK4b^, p21^CIP1^, and p27^KIP1^ [66]. One mechanism by which TGF-β stimulates p15^INK4b^ and p21^CIP1^ expression involves their transcriptional regulation, jointly controlled by the Smad complex and FoxO family transcription factors [67,68]. Another mode of cell cycle arrest is mediated by the Smad-dependent downregulation of c-Myc oncoprotein, a transcriptional repressor of the CDK inhibitors [64,69]. It is known that the G1 to S phase transition in the eukaryotic cell cycle is guided by the cooperation of CDK4 and CDK6 with cyclin D and CDK2 with cyclin E [70]. Through an elaborate pathway, these cyclin-CDK complexes progressively phosphorylate the retinoblastoma protein (pRb) during the G1 phase and trigger the release of the transcription factor E2F, which regulates the expression of genes required for the S phase transition. The increase in p15^INK4b^ and p21^CIP1^ levels by TGF-β critically thwarts the activities of cyclin D-CDK4/6 and cyclin E-CDK2 complexes, respectively, leading to pRb hypophosphorylation and the subsequent inhibition of E2F activity [71]. It is worth noting that these responses are specific to well-differentiated hepatoblast-like HCC cell lines, while poorly-differentiated mesenchymal-like HCC cell lines are generally resistant to TGF-β-mediated cytostasis [64,72]. In addition, TGF-β may, potentially, reduce cyclin D expression while increasing hypophosphorylated pRb levels via a Smad-interacting β-spectrin adaptor protein, SPTBN1, formerly known as embryonic liver fodrin or ELF [73].

TGF-β can also induce G2 arrest in HCC cell lines. The study by Hashimoto et al. described a form of G2 arrest in a pRb-deficient hepatoblastoma-like Hep3B cell line that was induced by p21^CIP1^ and p27^KIP1^. In terms of mechanism, these effects were associated with decreased expression of cyclin A and cyclin B and inhibition of cell division cycle 2 (CDC2) by Wee1 kinase, a regulator of the G2 checkpoint [74]. Recently, TGF-β-mediated stimulation of Hippo signaling, concomitant with increased expression of LATS1 and nucleus-to-cytoplasm translocation of YAP1, has been implicated in cell growth inhibition in HCC cells. Yet, linking these findings to cell cycle modulation constitutes an important agenda for future research [75].

Finally, a number of in vitro studies in hepatoma cells have revealed that TGF-β treatment may either dampen the expression of Id1 or induce the expression of CXXC5 transcription factors to promote TGF-β signaling and suppress cancer cell proliferation [76,77]. According to these studies, it is safe to conclude that TGF-β is a strong inhibitor of cell cycle progression in signaling-competent HCC cell lines.

#### 5.1.2. Cellular Senescence

Cellular senescence is an intrinsic defense mechanism designed to inhibit cell growth or division that can be triggered in response to a wide range of stressors, including genotoxic insults, oncogenic signaling, mitogens, and telomere attrition [78]. Having been formally described by Hayflick in the early 1960s, senescence has since been observed in diverse contexts, bringing new insights into the molecular understanding of this physiological and dynamic process during embryonic development, tissue remodeling, and aging [79]. It is important to appreciate that senescence is also a protective barrier against tumor development and progression, thwarting chronic and often uncontrolled cell proliferation and malignant evolution [78,80]. Arguably, senescence is a permanent withdrawal from the cell cycle in the G1 and, possibly, G2 phases [81]. Yet, reversible forms of cancer cell senescence have recently been described [78,82]. Cells undergoing senescence are characterized by distinct morphological features, such as enlarged cytoplasm, senescence-associated β-galactosidase activity (SA-β-Gal), the absence of proliferation markers, and a unique secretome signature, a phenomenon known as the senescence-associated secretory phenotype (SASP) [78,83]. The initiation and maintenance of cellular senescence is largely mediated by the complementary crosstalk of the cell fate regulatory circuits p53/p21^CIP1^ and pRb/p16^INK4a^, although p53- and p16^INK4a^-independent senescence has also been described [64,84].

In the case of the liver, cellular senescence has been implicated in chronic hepatitis, cirrhosis, and HCC [85,86]. Hepatocellular senescence has been widely studied in animal models of liver cancer. A groundbreaking study that exploited a chimeric HCC mouse model, in which p53-deficient embryonic hepatoblasts were transduced with an oncogenic Ras and injected into the livers of athymic mice, has elegantly illustrated that reactivating endogenous p53 results in a robust senescence response and tumor regression [87]. Yet, other observations with a murine model of intrahepatic Ras expression found that the immune-mediated clearance of premalignant senescent hepatocytes could suppress liver cancer development [88]. Therefore, consistent with the proposed role of p53 in premalignant tumors, the loss of p53-mediated surveillance mechanisms could permit evasion of cellular senescence and promote progression into a malignant phenotype [84,89].

TGF-β signaling has been recognized as a quintessential inducer of growth arrest in a variety of epithelial cell types [79]. In the case of liver physiology, TGF-β tonically regulates cell-autonomous mitogenic phenotypes and limits hepatocyte proliferation during acute liver regeneration following partial hepatectomy [90,91]. There is now evidence highlighting the preeminent importance of senescence in neoplastic cells, and malignant hepatocytes are no exception to this [92]. We previously demonstrated senescence induction by TGF-β in well-differentiated hepatoblast-like HCC cell lines (e.g., Huh7, Hep3B, and HepG2). The senescence morphology was distinguished by a strong SA-β-Gal signal and G1 arrest with augmented expression of p21^CIP1^ and p15^INK4b^ and a reciprocal decrease in phospho-pRb and c-Myc levels. This process was p53- and p16^INK4a^-independent and was attributed to TGF-β-mediated NADPH oxidase 4 (NOX4) activation and the accumulation of reactive oxygen species (ROS). Finally, in vivo administration of recombinant TGF-β1 yielded a dramatic senescence activity, coupled with a strong antitumor response in xenograft models [64]. These findings call attention to the utility of targeted prosenescent therapy, at least in certain HCC tumors with an intact TGF-β and senescence axis.

Consistent with our findings, Yoon et al. reported iron chelation-induced senescence in the Huh7 and Hep3B cell lines, which was associated with TGF-β-mediated p27^KIP1^ expression and G1 arrest [93]. Similarly, Sun et al. found that silencing Glypican 3, a member of the heparan sulfate proteoglycans, in the Huh7 and HepG2 cell lines provoked senescence and significantly decreased cell proliferation in vitro and suppressed tumor growth in vivo. These responses were linked to augmented TGF-β2 levels and upregulation of p21^CIP1^ and p15^INK4b^ [94]. Adding to our understanding of the contextual modulation of senescence, studies have established a role for paracrine TGF-β signaling in the non-cell-autonomous propagation of the senescence phenotype in normal and neoplastic hepatocytes. In other words, the state of SASP, derived from senescent cells, which are ostensibly rich in TGF-β, can help transmit a bystander senescence program to neighboring cells in a paracrine fashion, thereby interfering with HCC development [95,96].

Another dimension of complexity in this context is the causal relationship between replicative senescence and hepatocyte-specific telomere shortening [97]. Studies in human clinical samples of chronic liver diseases indicate that a gradual shortening of telomeres and the accumulation of senescent hepatocytes correlate with disease stage, implying that replicative senescence could potentially have an antitumor effect in multistep hepatocarcinogenesis [92,98]. However, caution must be exercised here, provided that reactivation of telomerase activity, a hallmark of advanced malignancies, may promote evasion from replicative senescence and facilitate neoplastic progression [99,100]. Yet, no doubt exists that canonical TGF-β/Smad signaling is a negative regulator of telomerase expression in a variety of normal and malignant cells [101,102]. Given its central role in chronic liver diseases, it is, thus, conceivable that the TGF-β pathway may also partake in replicative hepatocellular senescence, which merits further investigation [103].

#### 5.1.3. Autophagy

Autophagy represents an evolutionarily conserved intracellular mechanism that is essential for the regulation of vital biological processes and the careful maintenance of cell and tissue homeostasis. Under antagonistic conditions, the autophagic program seems to fine-tune cell fate decisions by recycling cytosolic resources and eliminating misfolded proteins and malfunctioning organelles [104]. This is especially true when cells encounter physiological stressors in their environment and intracellular milieu. In accordance with this, autophagy dysregulation causes the progressive accretion of damaged macromolecules and organelles, culminating in pathophysiological conditions, such as cancer [105].

As to the liver, multiple lines of evidence attest to a functional involvement of autophagy in balancing hepatic metabolism and protecting the organ against stress responses [106]. Data from experimental animal models indicate that autophagy machinery elicits cytoprotective cues against liver injury and uncontrolled cell death by selectively eliminating abnormal organelles and proteins [107]. Autophagy deficiency, on the other hand, causes an accumulation of protein aggregates, oxidative stress, DNA damage, and genomic instability, all of which can lead to chronic liver dysfunction and liver cancer [108,109]. This concept is exemplified in the study by Takamura et al., wherein genetically engineered mouse models with deletions of autophagy-related genes (ATG5 and ATG7) had impaired autophagy and developed benign tumors in the liver, suggesting that an intact autophagy program in mice may suppress inappropriate proliferation in the liver [110]. In addition, mice bearing a heterozygous disruption of Beclin1, an essential autophagy gene, exhibit increased susceptibility to spontaneous tumorigenesis in the liver, lung, and lymph tissues [109,111]. A central mechanism in this phenotype is the upregulation of p62/Sequestosome-1 (SQSTM1), an adaptor protein known to regulate NF-κB activation during tumorigenesis [112]. These principal findings were strengthened further by the loss of function changes observed in autophagy-related genes, in particular a monoallelic deletion of the tumor suppressor gene Beclin1, in multiple tumor types, including HCC [113,114]. Recognizing the link between autophagy and tumor suppression, the complex web of antineoplastic programs ascribed to autophagy are coupled to cell cycle arrest, senescence, and apoptotic and necrotic cell death [115,116,117,118].

Research over the past few decades has shown that TGF-β signals play critical yet contextually diverse roles in autophagy regulation. Early studies with kidney, heart, and mammary epithelial cells have yielded a growing list of biological responses, such as modulation of the extracellular matrix (ECM) and fibrogenic responses, as well as apoptosis and cell survival [119,120,121]. Others have identified that the TGF-β and autophagy axis contributes to liver fibrosis by activating hepatic stellate cells via the ERK and JNK signaling pathways [122]. Along these lines, a tumor-suppressive link exists between TGF-β signaling and autophagy machinery [123,124]. Molecular studies in the Huh7 HCC cell line and MDA-MB-231 mammary carcinoma cells have implicated both TGF-β signaling and the JNK cascade in autophagy induction, most likely by increasing the expression of several autophagy-related genes, Beclin1, ATG5, ATG7, and death-associated protein kinase 1 (DAPK). As part of this autophagic program, autophagosomes envelope long-lived proteins and fuse with lysosomes to cause degradation. More importantly, the circuit between TGFβ signaling and autophagy provokes apoptosis in Huh7 cells by modulating the expression of proapoptotic Bcl-2 family members, Bmf and Bim [125]. More recently, a distinct mechanism of autophagy induction has been described which, in particular, entails the activation of Unc-51-like kinase 1 (ULK1) by the crosstalk between TGF-β signaling and the TAK1-TRAF6-p38-MAPK pathway [126]. Whether this mode of autophagy activation induces apoptosis remains to be elucidated.

These findings, however, contrast starkly with others that have reported autophagy as a promoter of cell survival and tumor progression, underscoring a bidirectional and paradoxical role for autophagy machinery in cancer [127,128]. Along the same lines, TGF-β signaling and autophagy interactions in cancer may also be context-dependent. A few studies in HCC, mammary carcinoma, lung cancer, and glioma cell lines have already established a role for TGF-β-induced autophagy in exacerbating the aggressive traits of neoplastic cells [129,130,131]. When viewed in this light, this clearly suggests that once the antineoplastic mechanisms of TGF-β are compromised or autophagy activation is limited, the TGF-β signaling and autophagy axis may potentiate tumorigenic activities [131].

#### 5.1.4. Apoptosis

In addition to the cytostatic constraints, TGF-β is a well-known inducer of programmed cell death by apoptosis. Based on the knowledge accumulated over the last few decades, there is now a coherent understanding of the apoptotic aspects of TGF-β signaling in homeostasis and pathophysiological conditions. It is known that, like autophagy, TGF-β-induced apoptosis partakes in the selective elimination of excessive cells during developmental processes, such as limb formation and mammary gland involution [132,133]. Additionally, TGF-β can promote the homeostatic clearance of damaged or aberrant cells from a variety of normal tissues and organs, such as the immune system and the liver [91,134,135]. As exemplified in transgenic animal models, the TGF-β and apoptosis axis is implicated in governing liver size and regeneration in normal and regressing livers, as well as maintaining the stability of hepatocyte number in fibrosis and cirrhosis [136,137]. Similarly, selective overexpression of TGF-β in hepatocytes reveals massive apoptosis, as does short term treatment of cultured hepatocytes with TGF-β [137,138].

At the molecular level, cell death decisions by TGF-β are coupled to the activation of a complex network of genes and intracellular signaling circuits. Early research in liver-derived cell types identified the caspase-dependent cleavage of cellular inhibitors of apoptosis (cIAPs) and BAD, the Bcl-2-associated agonist of cell death, and the downregulation of the anti-apoptotic Bcl2 family member Bcl-xL as key mediators of the intrinsic apoptosis pathway [139,140,141,142]. Within the same context, transcriptional activation of the TGF-β-inducible early response gene 1 (TIEG1), a krüppel-like zinc finger transcription factor, facilitates proapoptotic actions by mitigating the expression of Bcl-2 [143,144]. Complementing these in vitro studies, liver-specific expression of Smad3 attenuates hepatocarcinogenesis by promoting apoptosis via Bcl-2 downregulation, strengthening the in vivo functional link between canonical TGF-β signaling and apoptotic machinery [144,145]. Along the same lines, TGF-β potentiates mitochondria-dependent cell death by upregulating Bmf and Bim in a Smad4- and p38-MAPK-dependent manner [146]. Interestingly, the proapoptotic actions of Bim are enhanced by Smad-dependent MAPK phosphatase 2 (MKP2) expression and the subsequent attenuation of ERK signaling, which, in aggregate, lead to a post-translational stabilization of Bim protein [147]. Later studies also linked DAPK, an immediate early response gene of TGF-β signaling, to mitochondrial-based proapoptotic responses [148,149]. Similarly, the immediate early activation of growth arrest and DNA damage-inducible 45β (GADD45β) by TGF-β triggers apoptosis in hepatocytes via a delayed activation of the p38-MAPK cascade [150]. In the same fashion, Daxx, an adaptor protein tied to Fas-induced cell death, has been connected to apoptosis in normal hepatocytes and p53-deficient hepatoma cells. Mechanistically, the phosphorylation of Daxx by homeodomain-interacting protein kinase 2, HIPK2 serves to propagate apoptotic signals from TGF-β receptors to the downstream effector JNK [151,152]. Aside from these, TGF-β can activate apoptotic events by stimulating the expression of programmed cell death 4 (PDCD4), a novel tumor suppressor gene that is frequently inactivated in HCC [153,154].

In addition to canonical TGF-β signaling, the execution of apoptosis may also entail the inhibition of the PI3K/Akt pathway through Smad-dependent upregulation of inositol phosphatase SHIP expression, which hydrolyzes phosphates in PIP3 and inhibits Akt signaling [49,155]. In a similar fashion, TGF-β can also promote apoptosis by activating JNK and p38-MAPK signaling through the TRAF6–TAK1 axis that may involve upregulation of XIAP [156,157]. Perhaps more important, c-Src, a cellular homologue of the Rous sarcoma virus-transforming gene, regulates programmed cell death by modulating both the JNK and p38-MAPK pathways [158]. In light of these findings, it is conceivable to conclude that these two pathways are integral to the TGF-β-induced cell death program in hepatoma cell lines.

To make the situation even more complex, TGF-β can disseminate proapoptotic signals through reactive oxygen species [159,160]. This process is generally related to decreased antioxidant gene expression (e.g., superoxide dismutases, catalase, and glutathione peroxidases) or increased NOX4 levels, which eventually result in Bcl-xL downregulation, cytochrome c release from the mitochondria, apoptosome formation, and caspase activation [138,161]. Lastly, an indirect route of apoptosis stimulation by TGF-β has also been described in hepatoma cells. The underlying mechanism works by an autocrine loop that requires the transcriptional induction of the TNF-related apoptosis-inducing ligand (TRAIL) by Smad-dependent signaling and Jun-Fos heterodimeric AP-1 transcription factor [162].

In summary, apoptosis is a paramount component of the TGF-β cell fate decision program in normal physiology and pathological conditions of the liver. Unless perturbed, the apoptotic circuit can be successfully evoked by conveying signals from both the canonical and non-canonical TGF-β pathways to intracellular effectors of the cell death machinery. In that manner, apoptosis represents yet another constraint that needs to be bypassed during tumor development.

### 5.2. Dysregulation and Loss of the Tumor-Suppressive Functions of TGF-β Signaling

It is widely accepted that escaping the powerful homeostatic instructions imposed by TGF-β signaling may confer a selective advantage to cancer cells. Accordingly, the functional dysregulation of TGF-β signaling in cancer is regarded as a tumorigenic event wherein tumor cells evolve a variety of strategies to effectively circumvent or subvert its antiproliferative defenses [59]. Genetic alterations and deregulated expression of TGF-β signaling mediators, regulators, and effectors are among the common mechanisms [163]. A recent study identified genetic abnormalities impacting 43 TGF-β superfamily genes in 39% of 9125 tumor samples across 33 TCGA (The Cancer Genome Atlas) tumor types [25]. Corroborating these findings, at least one of the 32 TGF-β superfamily genes was found to be commonly altered (present in about 40% of samples) in a cohort of 202 TCGA HCC tumors, emphasizing the high prevalence of genetic perturbations in the entire superfamily in HCC [164]. Intriguingly, however, unlike the high frequency of genetic aberrations in malignancies of the gastrointestinal tract, including those of the stomach, colon, and pancreas, the landscape of hotspot somatic mutations in the canonical TGF-β/Smad pathway remains relatively infrequent in HCC [25,165,166,167]. From a statistical perspective, the frequency of somatic mutations in TGF-β ligands and receptors and Smad genes is less than 1.0% (the cumulative pathway alteration rate is about 5%) in the TCGA cohort of HCC samples. This leads, in turn, to the conclusion that corruption of TGF-β signaling components during malignant conversion may occur through a collection of other abnormalities [167]. In support of this notion, TGFβRII and Smad4 are frequently inactivated by hypermethylation and reduced expression [168,169]. Other avenues of research have revealed the internalization of TGF-β receptors from the plasma membrane by a clathrin-dependent pathway and caveolae lipid rafts as an important regulatory mechanism in Smad signaling, whose deregulation has been linked to HCC development. As such, Caveolin-1, the primary component of the caveolae lipid rafts, impedes TGF-β growth inhibitory responses in HCC tumors and cell lines. More importantly, increased expression of Caveolin-1 has been associated with higher invasive capacity and metastatic phenotype, and poor prognosis in HCC [170,171].

Advances in the high throughput analyses of cancer genomes have contributed significantly to deciphering the mechanisms of TGF-β dysregulation in liver tumors. In a pioneering study, Coulouarn et al. managed to categorize human HCC tumors and cell lines by the presence of distinct expression signatures of early or late TGF-β-responsive genes. Interestingly, tumor cells bearing early TGF-β signature were selectively enriched for growth inhibitory responsiveness, whereas HCC subsets with the late TGF-β signature preferentially displayed an upregulation of positive cell cycle regulators (e.g., cyclins and CDKs), and genes pertaining to the invasive phenotype and EMT. Furthermore, the late TGF-β signature accurately predicted an aggressive tumor phenotype in vivo and correlated with a poor prognosis characterized by shortened patient survival and increased tumor recurrence [172,173]. These findings were further supported by a comprehensive analysis of transcriptional signatures that uncovered recurrent deregulations in the TCGA HCC cohort. Unsupervised hierarchical clustering of transcriptome data produced four distinct clusters with unique TGF-β activity signatures: inactivated, normal, activated, and highly activated. Interestingly, patients in the inactivated cluster demonstrated a loss of TGF-β tumor-suppressive functions and experienced shorter survival times than those in the normal or activated TGF-β cluster [164]. The same analysis found increased expression of Smad7 in the activated signature, which is consistent with a recent report demonstrating Smad7-mediated inhibition of nuclear Smad signaling contributes to liver carcinogenesis, most likely by activating the YAP/TAZ and Notch signaling cascades [174,175,176].

A diverse array of defects affecting the modulators, regulators, and effectors of TGF-β signaling have been documented. These defects seemingly mitigate antitumorigenic responses and contribute to HCC development. The SPTBN1 gene, which encodes a Smad adaptor β-spectrin protein that modulates nuclear Smad signaling, is the most frequently mutated gene, accounting for 4–6% of the TCGA HCC cohort [164,167]. Mice deficient in SPTBN1 develop spontaneous HCC tumors associated with elevated expression of cyclin D1, Cdk4, c-Myc, and MDM2, an oncoprotein that blocks p53 [73,177]. SPTBN1 is also impaired in HCC cell lines through genetic alterations and reduced expression [178]. Mechanistically, SPTBN1-deficient cells exhibit subcellular mislocalizations of Smad3 and Smad4, accompanied by the absence of Smad-dependent transcription and cytostatic responses [179].

Modulators and effectors of proapoptotic and cytostatic responses are also dysregulated in HCC (Table 2). Inhibition of caspases by anti-apoptotic proteins, such as Bcl-xL, Mcl-1, cIAPs, XIAP, and Survivin, is a frequent phenomenon in HCC tumors and cell lines [180]. It is known that the aberrant activation of NF-κB signaling upregulates the expression of these prosurvival molecules and provides enabling cues against TGF-β-mediated cell death in hepatoma cells [181,182]. In addition, cFLIP, an intracellular inhibitor of caspase-8 activation, which is abundantly expressed in HCC tissues and cell lines, is a critically linked effector of NF-κB-mediated resistance to TGF-β [183,184]. In addition to the activation of prosurvival genes, functional dysregulation of proapoptotic proteins, such as Bax and Bid, by subcellular mislocalization or downregulation, revokes TGF-β cell death programs and contributes to HCC development and progression [185,186].

As alluded to earlier, the transcriptional activities of TGF-β effector molecules are regulated by cofactors [187]. In this context, Smad-interacting transcriptional corepressors, such as Zeb2, Hey2, and serum response factor (SRF), whose expression is upregulated in HCC tumors and cell lines, can neutralize the TGF-β-induced transcriptional stimulation of p15^INK4b^ and p21^CIP1^, as well as the suppression of c-Myc [8,188,189,190]. In addition, the Ski/SnoN family of oncoproteins, which interact with R-Smads and negatively regulate TGF-β signaling, have also been implicated in HCC [191]. Much like these, EVI1, a zinc finger oncoprotein that is amplified and overexpressed in a considerable fraction of HCC cell lines and primary HCC tissues, interacts with Smad3 and antagonizes the TGF-β growth suppressive effects in HCC cells [192,193].

**Table 2:** Common dysregulation mechanisms affecting mediators, regulators, and effectors of TGF-β signaling in HCC.

Mediator, Regulators, and Effectors	Functions	Dysregulations	Reference
TGFβRII	TGFβRII transmits signals from the cell surface into the cell and is inactivated by hypermethylation in HCC.	Reduced expression in HCC	[168]
Smad4	Smad4 is the main effector in the Smadpathway and is inactivated by hypermethylation in HCC.	Reduced expression in HCC	[169]
Caveolin-1	Caveolin-1 blocks the TGF-β growth-inhibitory responsiveness.	Increased expression in HCC	[170,171]
Smad7	Smad7 interferes with R-Smad complex formation and antagonizes nuclear TGF-β signaling.	Increased expression in HCC	[174,175,176]
SPTBN1	SPTBN1 encodes a Smad adaptor β-spectrin protein that modulates nuclear TGF-β/Smad signaling.	Reduced expression in HCC	[164,167,178]
cFLIP	cFLIP is an intracellular inhibitor of caspase-8 activation and a preeminent modulator of NF-κB signaling.	Increased expression in HCC	[183,184]
Bax	Bax is a key regulator of the intrinsic pathway of apoptosis and mediates permeabilization of the outer mitochondrial membrane.	Subcellular mislocalization or reduced expression in HCC	[185]
Bid	Bid induces permeabilization of the outer mitochondrial membrane.	Subcellular mislocalization or reduced expression in HCC	[186]
Zeb2	Zeb2 is a Smad-interacting transcriptional corepressor.	Increased expression in HCC	[194]
Hey2	Hey2 is a Smad-interacting transcriptional corepressor.	Increased expression in HCC	[189]
SRF	SRF is a Smad-interacting transcriptional corepressor.	Increased expression in HCC	[188]
SnoN	SnoN is a regulator of TGF-β signaling that binds to the N-terminus of R-Smads.	Abundant expression in HCC	[191]
Ski	Ski is a regulator of TGF-β signaling that binds to the N-terminus of R-Smads.	Abundant expression in HCC	[191]
EVI1	EVI1 interacts with Smad3 and blocks TGF-β signaling.	Overexpressed in HCC	[192,193]
FHL	FHL proteins are tumor suppressor proteins that act as interaction partners of Smad molecules.	Epigenetically repressed in HCC	[195,196]
CXXC5	CXXC5 enhances TGF-β signaling by forming a positive feedback loop.	Reduced expression in HCC	[77]
KLF17	KLF17 enhances TGF-β signaling by forming a positive feedback loop	Reduced expression in HCC	[197]
Grk2	GRK2 phosphorylates the Smad3 linker region and blocks TGF-β target gene expression.	Increased expression in HCC	[198]
Axl	Axl causes aberrant phosphorylation of the Smad3 linker region and enhances TGF-β resistance.	Increased expression in HCC	[199]
p15^INK4b^	p15^INK4b^ plays a role as a cell growth regulator that impedes cell cycle G1 progression.	Deletion or promoter methylation in the INK4 locus	[72,200,201]
p16^INK4a^	p16^INK4a^ plays a role as an inhibitor of CDK4 and CDK6.	Deletion or promoter methylation in the INK4 locus	[72,200,201]

Four-and-a-half LIM (FHL) tumor suppressor proteins function as physical and functional interaction partners of Smad molecules. In terms of molecular mechanism, FHL proteins can phosphorylate and induce nuclear translocation of R-Smads in a casein kinase 1 delta-dependent but TGF-β receptor-independent manner, resulting in the upregulation of p21^CIP1^ and the suppression of c-Myc. Further evidence suggests that FHL genes are epigenetically silenced in HCC tumors and cell lines, and this correlates with impaired TGF-β responses [195,196]. As discussed earlier, the FoxO–Smad complex can block the G1/S transition by regulating the activities of the cyclin D and CDK axis. Numerous studies indicate that activated PI3K/Akt signaling phosphorylates FoxO transcription factors and prevents FoxO–Smad complex formation, effectively blunting the cytostatic responses of TGF-β [68,202]. Finally, the CXXC5 and KLF17 transcription factors potentiate TGF-β signaling and cytostatic responses via a positive feedback loop. Clinically, CXXC5 and KLF17 expression is significantly reduced in advanced HCC tissues, supporting the loss of this regulatory loop during HCC progression [77,197].

Recent analyses of genomic resources have revealed aberrant activation of anti-apoptotic and survival signaling cascades in HCC tumors [164,167]. While these enabling signals modulate non-canonical responses, their extensive crosstalk with TGF-β signaling has also been implicated in perturbing Smad-dependent transcriptional regulation and growth suppressive activities. A prime example is PDGF signaling, which promotes hepatocarcinogenesis while protecting tumor cells from TGF-β-induced growth arrest and apoptosis by converging the PI3K/Akt pathway with nuclear β-catenin signaling [203]. Consistent with this, EGFR signaling counteracts the proapoptotic effects of TGF-β in normal hepatocytes and HCC cells, in a large part by blocking TGF-β-induced NOX4 upregulation and ROS accumulation via the clathrin- and PI3K/Akt-dependent pathway [204,205,206].

Epigenetic mechanisms, such as chromatin remodeling and DNA methylation, modulate TGF-β signaling and contribute to the initiation and progression of cancer-associated processes, including hepatocarcinogenesis. Accumulating evidence suggests that epigenetic modifications in TGF-β signaling mediators, regulators, and effectors may result in the dysregulation of TGF-β/Smad signaling and in defective cytostatic responses. In this regard, a common mechanism that interrupts TGF-β-induced cytostasis is the inactivation of the INK4 locus (encodes p15^INK4b^ and p16^INK4a^) by deletion or epigenetic silencing via promoter methylation [72,200,201]. Another mechanism that impairs the antiproliferative effects of the TGF-β pathway in HCC is based on tristetraprolin (TTP), a negative post-transcriptional regulator of c-Myc with downregulated expression in HCC tumor tissues. Mechanistically, epigenetic silencing of TTP in HCC cell lines increases the half-life of c-Myc and confers resistance to TGF-β [207]. Similarly, Zhu et al. found that epigenetic silencing of human dachshund homolog 1 (DACH1) in HCC tumors and poorly-differentiated mesenchymal-like HCC cell lines is associated with dysregulated TGF-β signaling and the loss of transcriptional Smad responsiveness of the c-Myc and p21^CIP1^ genes [208]. According to a recent study by Bevant et al., exposing a mesenchymal-like HCC cell line SNU-449 to decitabine, a clinically relevant demethylating agent, impairs transcriptional responses and suppresses the expression of critical modulators of tumor-suppressive canonical TGF-β signaling (e.g., Serpine1, Smad4, TGF-β1, and Col1a1) while augmenting the levels of EMT-related transcription factors (e.g., Snail, Slug, Zeb1, and Zeb2) [209]. Collectively, epigenetic alterations may dysregulate TGF-β/Smad signaling in HCC, resulting in the loss of the tumor-suppressive effects of TGF-β while facilitating its tumor promoting actions.

### 5.3. Functional Switch of TGF-β Signaling in HCC

Over the past 20 years, a significant amount of experimental evidence has been collected showing that the dichotomous role of TGF-β signaling is modulated via the balance between the Smad3 linker and C-terminal phosphorylation [210]. A large amount of clinical data agree on a positive correlation between abundant Smad3 linker phosphorylation (coupled with limited C-terminal phosphorylation) and cancer stage, emphasizing that C-terminal phosphorylation may play an antiproliferative role during carcinogenesis, whereas linker phosphorylation emits prooncogenic signals [211,212].

Acting as negative-feedback loops for the tumor-suppressive arm of C-terminal Smad3 signaling, oncogenic pathways counteract senescence- or apoptosis-inducing circuitry by linker region phosphorylation [213,214]. More importantly, this process often works as a molecular switch, enhancing the mitogenic effects of survival pathways and contributing to the tumor promoting arm of TGF-β. A prominent example encompasses Smad3 linker region phosphorylation by activated JNK or oncogenic ERK signaling, which functionally interferes with TGF-β cytostatic actions by suppressing p21^CIP1^ induction while also converting Smad3 signaling to an oncogenic pathway via the transcriptional activation of c-Myc [215,216,217,218]. Yet another signaling node that antagonizes C-terminal Smad3 activity involves the G protein-coupled receptor kinase Grk2-mediated linker phosphorylation. Acting in a negative feedback mechanism, Grk2 diminishes TGF-β-induced target gene expression and growth suppression in HCC cells [198]. Similarly, a few studies identified Axl receptor tyrosine kinase as a mediator of TGF-β resistance through aberrant Smad3 linker phosphorylation. In fact, Axl switches Smad3 signaling towards dedifferentiation and invasion of HCC cells by upregulating prometastatic target genes and creating a pro-tumorigenic microenvironment via differential regulation of the chemokine CXCL5 [199,219]. Finally, compelling research convincingly shows that intracellular kinases, including CDK8 and CDK9, GSK-3β, as well as the p38-MAPK and Rho/ROCK cascades, also interfere with the cytostatic TGF-β circuit by phosphorylating R-Smads in their linker regions [210,220].

When portrayed in this way, genomic and transcriptomic perturbations in TGF-β signaling components, rewiring of the transcriptional responses of intracellular signaling circuitry, and defective cytostatic and proapoptotic processes may individually or collectively decouple TGF-β from tumor-suppressive outcomes and instead provide functional capabilities to switch the responses towards the activation of the pro-tumorigenic arm.

### 5.4. Tumor Promoter Role of TGF-β Signaling in HCC

**Figure 4:**
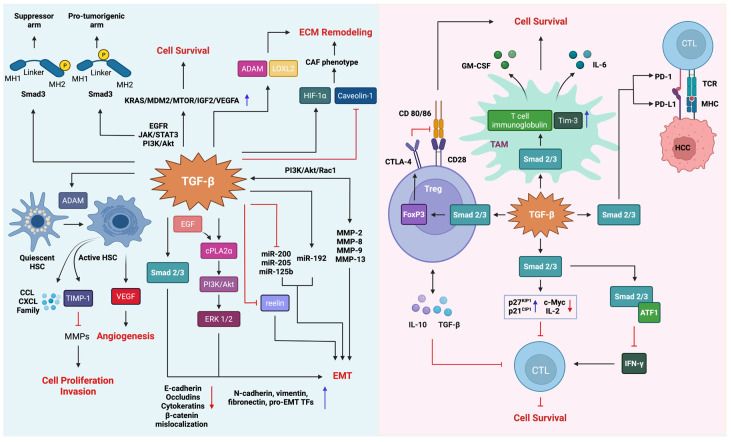
Pro-tumorigenic mechanisms of TGF-β signaling. The tumor promoter role of TGF-β signaling includes featured, such as cancer cell proliferation, epithelial–mesenchymal transition, remodeling of the tumor microenvironment, and tumor immune escape.

The pro-tumorigenic effects of TGF-β signaling are mediated by a highly complex set of mechanisms that affect the cancer cell itself and the tumor stroma [9,221,222]. As previously stated, a considerable fraction of HCC tumors and cell lines selectively lose sensitivity to TGF-β-mediated growth suppression, despite retaining an otherwise intact signaling cascade. Relieved from the cytostatic pressure, neoplastic cells can now respond to the same signal with distinct biological ends, including cancer cell proliferation, EMT, and invasion. In addition, reciprocal TGF-β signaling between the tumor cells and the stroma may promote a reprogramming of the microenvironment and create a milieu conducive to tumor growth, invasion, and metastasis. In principle, this is achieved by activating stromal cells, inhibiting the tumor-suppressive phenotype of immune cells, remodeling the extracellular matrix (ECM), and inducing angiogenesis [223,224] (Figure 4).

#### 5.4.1. Cancer Cell Proliferation

A compelling body of evidence indicates that after losing sensitivity to the antitumor effects, malignant hepatocytes may respond to TGF-β with sustained survival and proliferation. Circumstantial support for this notion comes from comparative analyses of malignant lesions. HCC tumors with active TGF-β signatures display abundant expression of oncogenes (e.g., KRAS, MDM2, MTOR, IGF2, and VEGFA), strengthening the model that a functional link exists between tumor promoting TGF-β and oncogenic circuits [164]. This observation is consistent with the increasing amount of in vitro data in which elevated TGF-β levels in HCC cell lines coincide with the aberrant activation of survival programs. Notable among these survival factors are EGFR, JAK/STAT3, and PI3K/Akt, all of which are known to accelerate chronic proliferation in malignant hepatocytes [10,225].

#### 5.4.2. RNA Modifications

The initiation and progression of cancer is facilitated by various genetic, epigenetic, and epitranscriptomic mechanisms. Comprehensive and integrative molecular studies in recent years have implicated aberrant epitranscriptomic RNA modifications in the multistep progression of HCC. N6-methyladenosine (m6A) methylation is the most prevalent and abundant modification of eukaryotic mRNA transcripts [226]. The multicomponent methyltransferase complex, consisting of methylases, or writers, such as METTL3 and METTL14, and regulator proteins, such as METTL16, RBM15, WTAP, and ZC3H13, is responsible for installing m6A modifications, while FTO and ALKBH5, which function as demethylases, or erasers, catalyze the removal of methyl groups from m6A [227,228]. The YTH domain-containing family proteins (e.g., YTHDF1, YTHDF2, YTHDF3, YTHDC1, and YTHDC2), heterogeneous nuclear ribonucleoprotein family (e.g., HNRNPA2B1, HNRNPC, and HNRNPG), and IGF 2 mRNA-binding proteins (e.g., IGF2BP1, IGF2BP2, and IGF2BP3), or readers, recognize m6A modifications and regulate a spectrum of intracellular mechanisms. The deregulation of such epitransciptomic mechanisms has been shown to impair almost every step of mRNA processing, including translation, stability and degradation, alternative splicing, and nuclear transport, which collectively result in the dysregulation of critical signaling pathways (e.g., the TGF-β/Smad signaling cascade), which regulate cell proliferation, apoptosis, EMT, and invasion [229]. Using bioinformatics analyses on transcriptome and clinical data in the TCGA cohort of HCC samples, a recent study has shown that eleven genes related to m6A methylation, including METTL3, YTHDF1, YTHDF2, and FTO, were substantially elevated in HCC tumors. This study further underscored that some of these genes could be promising prognostic markers or therapeutic targets in HCC [230]. The study by Liu et. al. conducted a multi-omic analysis of METTL3 and METTL14 in HCC and identified that these two genes had opposite expression and prognostic values. The authors also investigated m6A-modified differentially expressed genes, biological processes, and signaling pathways regulated by METTL3 and METTL14 knockdown in the HCC cell line HepG2. Their analyses found that mRNAs whose stability or translation efficiency were governed by METTL3 and METTL14 jointly facilitate biological processes such as protein ubiquitination and the cell cycle, as well as multiple signaling pathways, including TGF-β signaling [231].

The emerging field of epitranscriptomics has been a subject of extensive research in recent years. We are yet to find many answers to the network of molecular mechanisms regulating mRNA modifications and their relation to the pathogenesis of HCC, which warrants further investigation. However, with the current understanding of recent research in the field, it is possible to conclude that m6A modifications and the TGF-β signaling axis are involved in a variety of complex downstream mechanisms that intrinsically contribute to the hallmarks of HCC, including increased cell proliferation, evasion of apoptosis, the induction of EMT, invasion, and metastatic pathways [232].

#### 5.4.3. Epithelial–Mesenchymal Transition and Tumor Microenvironment Remodeling

The epithelial–mesenchymal transition (EMT) is a cellular plasticity program hijacked by carcinoma cells to adapt to certain environmental cues or counteract cellular stress responses, such as apoptosis and senescence [233,234]. The acquisition of mesenchymal traits is accomplished by the loss of epithelial polarity and cell–cell contacts. This phenotype is generally followed by increased motility, invasion, and metastasis [235]. The most notable hallmark of this process is the loss of the epithelial marker E-cadherin, a central component of cell–cell adhesion junctions. Other molecular changes include the decoupling of β-catenin from adherens junctions and the diminished expression of cytokeratins and occludins, with a reciprocal increase in mesenchymal markers, such as N-cadherin, vimentin, and fibronectin [236]. Mounting evidence has established EMT as a key driver of liver cancer progression [10]. Studies in well differentiated human HCC tumors indicate that E-cadherin is localized to the plasma membrane, whereas poorly differentiated HCC tissues have cytoplasmic dislocation or a frequent loss of E-cadherin and nuclear accumulation of β-catenin, reflecting, in large part, the progressive nature of EMT during the course of multistep hepatocarcinogenesis [237,238].

TGF-β has long been known as a preeminent inducer of hepatocellular EMT. In malignant hepatocytes, TGF-β induces a downregulation of E-cadherin and other epithelial markers while concurrently increasing the expression of mesenchymal proteins, including N-cadherin and vimentin, as well as pro-EMT transcription factors, such as Snail, Slug, Twist, Zeb1, and Zeb2 [172,239]. Intriguingly, however, a few studies indicate that senescence or apoptosis may still be the dominant phenotype in certain settings. As such, inherently noninvasive HCC cell lines respond to TGF-β treatment with a partial EMT while continuing to express residual epithelial traits [61,136]. Arguably, these findings signify a concurrent competition between distinct cellular fates, which can be explained, in part, by the dynamic crosstalk between the signaling circuits radiating from growth factor receptors [240]. For example, TGF-β-induced EMT involves EGF-dependent activation of the PI3K/Akt pathway, which requires cytosolic phospholipase A2α (cPLA2α), a key enzyme that stimulates lipid mediator production [241]. Notably, however, cPLA2α may also counteract tumor-suppressive R-Smad signaling [242]. It is known that EMT programs can also be regulated by noncoding miRNAs [243]. Qualifying a common role for miRNAs in hepatocarcinogenesis, a number of miRNAs (e.g., miR-216a/217, miR-612, miR-125b, and miR-451) are reported to partake in hepatocellular EMT [244,245,246,247]. Besides, prominent miRNA families, such as miR-200, miR-205, and miR-192, have been linked to the TGF-β and EMT axis. These miRNAs evoke EMT by regulating the expression of pro-EMT transcription factors at the post-transcriptional level [248,249,250].

An emerging body of evidence suggests that m6A modifications can regulate TGF-β signaling, EMT phenotype, and cancer stemness. A recent study by Lin et al. has shown that EMT in HCC cells is regulated by the m6A modification levels of mRNAs. The m6A modification has been identified in a total of 128 genes related to cell association, adhesion, and migration in cells undergoing EMT. Specifically, TGF-β-induced Snail expression was triggered by an m6A modification that was sufficient to drive EMT-mediated cancer progression [232]. Another study by Wang et al. identified RALY RNA binding protein-like (RALYL), a liver-progenitor-specific gene, to be associated with strong tumorigenic activity, stemness, chemoresistance, and invasion in HCC cell line models, as well as poor prognosis and metastasis in clinical HCC patients. Mechanistic investigations revealed that RALYL-induced stemness, EMT, and invasion was linked to its interaction with and the enhanced stability of TGF-β2 mRNA by modulating m6A modification [251]. Similarly, METTL3-induced m6A modifications have also been implicated in the increased cell migration and expression changes of EMT-related genes, such as E-cadherin, fibronectin, and vimentin [252]. Molecularly, these effects were attributed to the regulation of the total mRNA levels and mRNA stability of JunB. Furthermore, recent research has shown that m6A modification of mRNAs can be directly modulated by the TGF-β signaling components. In this case, Bertero et al. demonstrated that Smad2/3 functionally interacts with the m6A methyltransferase complex, consisting of METTL3, METTL4, and WTAP, and promotes the binding of the complex to a subset of transcripts, which facilitates m6A deposition on specific regulators of cell fate decisions, such as pluripotency and stemness [253].

Multiple lines of evidence indicate that the properties of cancer stem cells (CSCs), or tumor-initiating cells (TICs), affect the malignancy of tumors, including factors such as recurrence and metastasis [254]. Several studies have shown that about half of the HCC tumors are clonal and are expected to be enriched with CSC populations. Besides, a longstanding mechanistic link exists between CSCs and TGF-β-induced EMT, which arguably contributes to the expansion of CSCs. CD133, a transmembrane protein also known as prominin-1, has been considered a putative stem cell marker in HCC [255]. An earlier study by You et al. confirmed that CD133+ HCC cells have stem cell features characterized by multilineage potential, increased proliferation, and tumorigenic capacity in vivo. More importantly, the study also showed that the expression level of CD133 could be upregulated by TGF-β via the Smad-dependent pathway in Huh7 cells, an HCC cell line known to respond to TGF-β with a robust senescence phenotype [64,256]. Recently, Malfettone et al. have shown that TGF-β-induced plasticity in epithelial-like HCC cells, characterized by a partial EMT phenotype, promotes a switch in the expression of stem-related marker genes from EPCAM or CD133 to CD44, accompanied by a switch to an enhanced migratory and invasive phenotype. The partial EMT phenotype further suggests that epithelial-like HCC cells display a higher stemness potential than mesenchymal-like cells [257].

A corollary phenotype tied to TGF-β-mediated EMT is cancer cell invasiveness. This phenotype is explained by the enhanced motility of cancer cells and their ability to invade the surrounding matrix by modifying the extracellular milieu, in particular the non-cellular ECM components [258]. Several effectors are engaged in ECM remodeling [259]. TGF-β can stimulate the expression of matrix degrading metalloproteinases (MMPs), in particular MMP-2, MMP-8, MMP-9, and MMP-13, and promote the migration and invasion of HCC cells [260,261,262]. Importantly, MMPs can reciprocally activate TGF-β through PI3K/Akt/Rac1 signaling, a critical positive feedback loop in tumor cell mesenchymalization and malignant evolution [261,263]. Activated TGF-β also triggers vascular invasiveness by modulating the expression or the functional status of integrin subunits [264,265]. Mechanistically, this is orchestrated by an EMT program that is mainly driven by the PI3K/Akt/Snail/PTEN axis and laminin-5 (Ln-5), a basement membrane glycoprotein that is associated with HCC progression [266,267]. Aside from these, Luo et al. has recently demonstrated that TGF-β enhances cell migration by downregulating reelin, a large secreted ECM glycoprotein whose dampened expression in HCC correlates with a high recurrence rate [268,269]. In addition, TGF-β promotes cytoskeletal remodeling, vascular mimicry formation, and local invasion in HCC by upregulating lysyl oxidase-like 2 (LOXL2), an enzyme that facilitates the crosslinking of ECM components, such as collagen and elastin [270,271].

In addition to the non-cellular components, the HCC microenvironment is composed of malignant hepatocytes and non-tumoral cell types, including fibroblasts, hepatic stellate cells (HSCs), endothelial cells, macrophages, and immune cells [9,272]. In this cellular milieu, paracrine and autocrine TGF-β signaling modulate bidirectional tumor-stroma interactions and support malignant cell dissemination [224,273]. It is increasingly apparent that cancer-associated fibroblasts (CAFs) are one of the major stromal cell types that favor aggressive traits in HCC progression [272]. By downregulating Caveolin-1 expression and activating HIF-1α, TGF-β acts as a critical activator of the tumor-supportive CAF phenotype [273,274]. Once activated, the resident CAFs provide important cues for tumor cell proliferation, invasion, and immune evasion, such as ECM deposition and the secretion of growth factors and cytokines (e.g., hepatocyte growth factor, Wnt family ligands, stromal cell-derived factor 1α, and IL-6) [222,275,276]. Much like CAFs, HSCs are also an important source of extracellular proteins and play fundamental roles in tumor evolution [276]. Experimental evidence indicates that tumor-derived TGF-β can feed signals to HSCs and induce the expression of ADAMs, a family of transmembrane and secreted proteins with metalloendopeptidase activity, and the secretion of tissue inhibitors of metalloproteinases (TIMPs), as well as the CCL and CXCL family of chemokines [277,278]. In this context, TIMP-1, a natural inhibitor of MMPs, can instigate heterotypic signaling between HSCs and HCC cells by activating FAK signaling in tumor cells, favoring proliferation and invasiveness [278]. The bidirectional interaction between HCC and activated HSCs may also sustain tumor growth and facilitate systemic spread through endothelial cell proliferation and neovascularization via vascular endothelial growth factor (VEGF) secretion [279,280]. Notably, vasculogenesis and angiogenesis by TGF-β require a rapid yet transient onset of apoptosis in endothelial cells orchestrated by the VEGF/VEGFR2 axis [281]. Finally, tumor-associated macrophages (TAMs) represent a critical cellular component that can infiltrate the tumor stroma and provide cancer-related proinflammatory and proangiogenic cues [282,283,284]. Taken together, the autocrine and paracrine interplay of TGF-β signaling with other tumor stromal cells constitutes a strong pro-tumorigenic property in HCC cell invasion and metastasis.

#### 5.4.4. The Role of TGF-β in Immune Suppression

TGF-β signaling acts as a key regulator of immune cell proliferation, differentiation, and development in the liver. Thus, TGF-β plays an integral role in the physiological control of hepatic immune homeostasis, ensuring a tight balance between immune tolerance and activation [285]. With this portrayal, dysregulated TGF-β signaling is significantly associated with the diminished antitumor activity of effector cells concomitant with the functional exhaustion of immune cells. The reciprocal communication between the tumor and the supporting stromal cells has been emphasized in immune evasion. Of particular relevance to this notion is that the stromal cells, including HSCs, CAFs, TAMs, myeloid-derived suppressor cells (MDSCs), natural killer (NK) cells, dendritic cells (DCs) and regulatory T cells (Tregs), can be both important sources of and responders to TGF-β [286].

It has been widely accepted that TAMs are characterized by an M2-like phenotype. Tumor-derived TGF-β stimulates M2 polarization of TAMs via Smad-dependent upregulation of T-cell immunoglobulin and mucin-domain containing protein-3 (Tim-3), a well-recognized negative regulator of T-cell-mediated responses. Tim-3 expression in TAMs enhances the production of major protumoral cytokines, including granulocyte-macrophage colony-stimulating factor (GM-CSF) and IL-6, which can impinge back on HCC cells, conferring sustained proliferation, migration, and invasion, alongside immunosuppression [287]. Notably, TGF-β also thwarts the proinflammatory phenotype of macrophages mediated by the TNF/NF-κB/Smad7 axis [288]. Scientific evidence suggests that HCC patients demonstrate high levels of tumor-infiltrating Tregs [289,290]. While suppressing most immune cells, tumor- and stroma-derived TGF-β promotes Treg activation via a Smad-dependent upregulation of a forkhead box protein 3 (FoxP3) transcription factor, together with amphiregulin and IL-2 [291,292]. An important fact for the Treg compartment is that its selective expansion and activation within the tumor stroma can induce the production of additional stromal modifiers, such as adenosine, arginase, and IL-10. This may then mitigate the effector functions of cytotoxic T lymphocytes (CTLs) and allow neoplastic cells to avoid CTL-mediated clearance [167,286]. Additionally, TGF-β can directly suppress CTL growth and differentiation through the upregulation of p21^CIP1^ and p27^KIP1^, and the reciprocal downregulation of c-Myc and IL-2 [293,294]. TGF-β similarly inhibits the antitumor activity of CTLs by dampening the expression of interferon gamma (IFN-γ) via the transcription factor ATF1 [163]. In addition to these, cancer-stimulated MDSCs can also partake in the expansion of Treg cells by abrogating hepatic NK cell activity through membrane-bound TGF-β and the secretion of IL-10 [286,295]. CAFs and DCs are also known to exploit TGF-β signals to deploy pro-tumorigenic actions by releasing several molecules that, in aggregate, attract Tregs to tumor sites to compromise CTL immune responses [283].

Adding to the sophisticated mechanism of Treg-mediated immune suppression, FoxP3 promotes the expression of membrane-bound CTLA-4, an immune checkpoint receptor, also known as CD152, to compete with the costimulatory molecule CD28 [296,297]. Similarly, TGF-β can stimulate the expression of programmed cell death protein 1 (PD-1) in tumor-associated CTLs, and its ligand PD-L1 in cancer cells. PD-1 engagement with PD-L1 counterbalances antigen receptor signaling and the secretion of immunosuppressive cytokines, thus inhibiting the proliferation of CTLs and causing their exhaustion [167,298]. Collectively, these lines of evidence strongly indicate that TGF-β signaling is a potent immunosuppressant in HCC, underscoring that the inhibition of TGF-β within the hepatic microenvironment might unleash the immune system against the tumor.

## 6. Therapeutic Implications of TGF-β Signaling

The identification of druggable molecular mechanisms encompassing the initiation and progression of HCC tumors is critical to developing novel targeted therapies. The majority of currently approved drugs are small molecule multi-kinase inhibitors of the VEGF family of receptors (VEGFR) and a number of receptor tyrosine kinases and monoclonal antibodies targeting immune checkpoint inhibitors [299]. Sorafenib, an oral multi-kinase inhibitor, has been considered an effective first-line standard of care for patients with unresectable HCC. Sorafenib inhibits several tyrosine kinases, including VEGFR, platelet-derived growth factor receptor (PDGFR), c-Kit, and Flt3, suppressing tumor cell proliferation, inducing apoptosis, and mitigating tumor angiogenesis [300]. Yet, Sorafenib responses may be limited, due to high levels of genetic heterogeneity or the development of acquired drug resistance, which often leads to HCC progression. The benefits of combining Sorafenib with other therapies are currently being investigated [301]. Lenvatinib is an oral multi-kinase inhibitor that inhibits tumor cell proliferation and angiogenesis by targeting the VEGFR, fibroblast growth factor receptors (FGFR), PDGFR, rearranged during transfection (RET), and c-Kit. Although it is not inferior to Sorafenib for overall survival, Lenvatinib is approved for the first-line treatment of unresectable HCC [302]. Recent data suggest that Lenvatinib, in combination with immune checkpoint inhibitors, has the potential to be a future therapeutic strategy for patients with advanced HCC [303]. Regorafenib, much like the other multi-kinase inhibitors, suppresses tumor cell proliferation, survival, and angiogenesis by targeting a range of receptor tyrosine kinases [304]. Its chemical structure is nearly identical to that of Sorafenib, except for an additional fluorine atom in the central phenyl ring. This structural difference is thought to provide a broader range of target kinases [305]. In fact, Regorafenib is an effective second-line treatment for advanced HCC patients who tolerated Sorafenib therapy but experienced tumor progression. Finally, Cabozantinib is a potent multi-kinase inhibitor that simultaneously targets multiple kinases, including VEGFR, MET, RET, Flt3, c-Kit, and Axl. The success of Cabozantinib clinical studies have expanded the range of multi-kinase inhibitors that can be used as a second-line treatment for advanced HCC patients previously treated with Sorafenib [306].

In addition to the pro-tumorigenic mechanisms cited above, TGF-β signaling has been linked to targeted therapy resistance and the clinical failure of immune checkpoint inhibitors, rendering this pathway a potentially druggable hallmark of HCC. This statement is justified by the considerable efforts invested in a wealth of basic research studies and clinical trials testing selective antagonists to block the TGF-β pathway for both the tumor and the microenvironment [299,307,308]. Within the context of therapy failures, the relationship between the TGF-β-induced EMT program and cancer stemness phenotype is becoming increasingly clear [234,309]. Increased expression of drug exporters, reduced cell proliferation, and evasion of apoptosis are all parts of the general mechanisms associated with EMT-mediated drug resistance [310]. Evasion of immune responses is another important mechanism by which EMT confers anticancer drug resistance by altering the expression of the molecules involved in immunosuppression or immune evasion [310,311]. In the context of Sorafenib resistance, for example, the karyopherin subunit alpha 3 (KPNA3)-Akt/Erk-Twist signaling axis has been shown to induce EMT and drive drug resistance [312]. TGF-β signaling has also been implicated in establishing Sorafenib resistance through increased expression of multiple receptor tyrosine kinases, as well as a CD44-associated mesenchymal-like phenotype [313,314]. Increased expression of CD44 with the concomitant acquisition of cancer stemness properties has been identified to impair Sorafenib-induced apoptosis in HCC cell lines [313]. Moreover, reduced expression of CD44, which arguably increases the expression of epithelial-related genes, concomitant with a decrease in stemness attributes, resensitizes HCC cells to Sorafenib treatment [315]. Finally, resistance to anticancer compounds has been linked to the transcriptional or post-translational regulation of EMT-related genes [316,317]. Targeting TGF-β for cancer therapy can be realized in three possible settings: the inhibition of ligand activity, ligand-receptor interaction, and intracellular signaling [318,319]. The current list of TGF-β signaling antagonists that advanced to HCC clinical trials includes soluble ligand neutralizing antibodies (e.g., SRK-181, SAR439459, NIS793, and Fresolimumab), rationally designed ligand traps (e.g., AVID200 as a TGF-β1/β3 trap), small molecule inhibitors of TGFβRI (e.g., Vactosertib and Galunisertib), and TGF-β2-targeting antisense oligonucleotides (e.g., Trabedersen) [308,320]. Table 3 compiles the complete information on molecules targeting TGF-β signaling components, including those mentioned above and others. Despite having a favorable tolerability profile in metastatic patients, translating these pharmacological interventions from bench to bedside has proven challenging, given the apparent heterogeneity of HCC tumors [321,322,323]. Besides, the clinical implications of the dual and opposing activities of TGF-β signaling are only beginning to be revealed. Nonetheless, the available clinical data accentuates that combining TGF-β antagonists with immune checkpoint inhibitors, in addition to monotherapy, represents a highly promising, safe, and effective therapeutic strategy.

Arguably, combinatorial therapies, at least for some patients, can revert the immunosuppressive environment into an immunosupportive milieu. In line with this scenario, the phase I study of Bintrafusp alfa, a first-in-class bifunctional fusion trap composed of a therapeutic antibody against PD-L1 fused to the extracellular domain of TGFβRII, in solid tumors including HCC suggests a manageable safety profile and preliminary efficacy [328]. In addition, ABBV-151, an antibody that selectively targets glycoprotein-A repetition predominant protein (GARP), a chaperone that instructs latent TGF-β1 for activation in Treg cells, is also being tested in combination with an anti-PD-1 antibody in a phase I clinical trial in patients with locally advanced or metastatic solid tumors. Currently, other therapeutic combinations of TGF-β antagonists with multi-kinase inhibitors, chemotherapeutic reagents, and radiotherapy modalities are also under clinical investigation [329]. For example, phase I and II combination studies of Galunisertib and Sorafenib, an oral multi-kinase inhibitor, revealed good tolerance and efficacy, suggesting that Galunisertib, in combinations with other therapeutic agents, might have a promising clinical potential [326,330]. In support of that, Galunisertib, in combination with Nivolumab, is currently being evaluated in a phase II clinical trial in advanced HCC [299].

A better understanding of the mechanistic details of how TGF-β switches from a tumor suppressor to a pro-tumorigenic signal during hepatocarcinogenesis and the identification of biomarkers that would help better stratify patient selection may foster the clinical development of more effective TGF-β antagonists and improve patient responses to combination therapies.

## 7. Conclusions

TGF-β signaling plays a dichotomous role in cancer, and HCC is no exception to that. After acting as a tumor suppressor in the early stages of cancer, as the malignancy evolves, TGF-β undergoes a functional diversion towards a pro-tumorigenic signal that impacts the acquisition of multiple aggressive tumor traits. Increased levels of the TGF-β cytokine in tumors and the stroma, combined with its pleiotropic nature, make the TGF-β pathway a promising therapeutic target in HCC. Clinical exploitation of TGF-β targeting typically aims to thwart its tumor promoting functions while limiting, if possible, unintended targeting of antitumor activities. Despite recent achievements in TGF-β therapeutics, further research is needed to correlate the differential attributes of TGF-β signaling with the success of its clinical inhibition. In that sense, the identification of reliable prognostic and predictive biomarkers would allow for better a stratification of patient selection. These efforts may also provide valuable insights into the clinical decisions to select those who would benefit the most from TGF-β targeting, alone or in combination with other antitumor agents.

## Figures and Tables

**Table 1:** The bright and the dark sides of TGF-β signaling in HCC.

Bright Side	Dark Side
Cellular Senescence	Cancer Cell Proliferation
G1 Cell Cycle Arrest	RNA Modifications
G2 Cell Cycle Arrest	Epithelial–Mesenchymal Transition (EMT)
Autophagy	Invasion and Angiogenesis
Apoptosis	ECM Remodeling

**Table 3:** Different strategies targeting TGF-β signaling components for cancer therapy.

Mode of Action	Target	Name	Type	References
TGF-β ligand inhibitors	TGF-β1, TGF-β2, and TGF-β3	Fresolimumab	Neutralizing antibody	[318]
	TGF-β1, TGF-β2, and TGF-β3	SAR439459	Neutralizing antibody	[320]
	TGF-β1, TGF-β2, and TGF-β3	NIS793	Neutralizing antibody	[320]
	TGF-β1 and TGF-β3	AVID200	Ligand trap	[308]
	TGF-β1	Metelimumab	Neutralizing antibody	[324]
	TGF-β2	Trabedersen	Antisense oligonucleotide	[308]
	TGF-β2	Lucanix	Vaccine	[321]
	TGF-β1	LY2382770	Neutralizing antibody	[325]
	TGF-β1	SRK-181	Neutralizing antibody	[321]
	TGF-β2	Lerdelimumab	Monoclonal antibody	[326]
	TGF-β1	Disitertide	Peptide	[326]
	TGF-β1 and TGF-β2	FANG Vaccine	Vaccine	[326]
	TGF-β1	ISTH0036	Antisense oligonucleotide	[325]
TGF-β receptor inhibitors	TGFβRI	Galunisertib	Small molecule inhibitor	[318]
	TGFβRI and TGFβRII	LY2109761	Small molecule inhibitor	[327]
	TGFβRI	PF-03446962	Monoclonal antibody	[318]
	TGFβRI	IMC-TR1	Monoclonal antibody	[320]
	TGFβRI	Vactosertib	Small molecule inhibitor	[318]
	TGFβRI and TGFβRII	P144	Ligand trap	[325]
